# Looking at the role of direct and indirect pathways in basal ganglia networks at different levels

**DOI:** 10.1186/1471-2202-16-S1-P225

**Published:** 2015-12-18

**Authors:** Rahmi Elibol, Neslihan Serap Şengör

**Affiliations:** 1 Electronics and Communication Engineering, Istanbul Technical University, Istanbul, Turkey

## 

The role of basal ganglia in motor action initiation and selection has been well studied and now it is evident that impairment in this structure causes not only causes movement disorders as Parkinson's disease, Huntington's disease but also behavioral dysfunctions as addiction, obsessive-compulsive disorder [1-4]. In order to understand the mechanisms giving rise to motor actions, cognitive processes related to these actions as decision making and the diseases occurring due to malfunctioning of these structures, various computational models of direct and indirect pathways have been proposed [5-9]. Here, in order to set a simple relation between models of basal ganglia at different levels, a simple mass model indicating the controversial role of direct and indirect pathways will be introduced first. While dopamine (DA) in direct pathway enhances the activity in Thalamus giving rise to inhibition of action, arise of DA in indirect pathway disinhibits Thalamus activity promoting the action to take place. This activity can be followed from Figure 1 for different DA levels. Based on the results of this simple mass model, spiking neural network (SNN) is built by point neurons and the relation between the local field potential of this SNN and simple mass model will be discussed. The aim is to build a simple relation between different levels of computational models which would help investigating the mechanisms behind the cognitive processes without engaging in detailed models initially. Thus, the simple mass model proposed would be primary model giving a chance to test the initial interpretation of the concepts formed and lead to setting up more detailed, realistic models.

**Figure 1:**
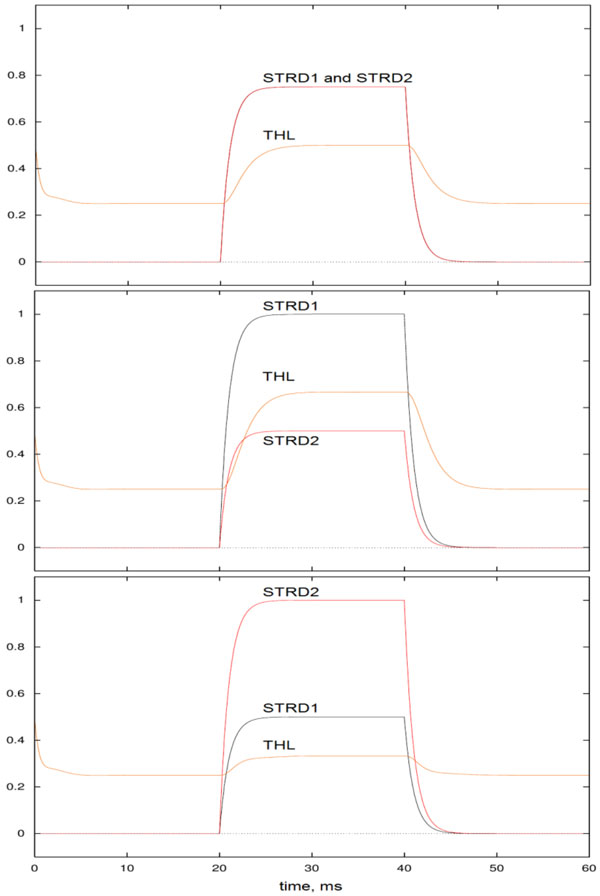
**DA level is normal, high and low at top, middle and bottom, respectively**. THL activity is normal, high and low similar to STRD1 activity and contrary to STRD2 activity.

## References

[B1] Kropotov J Etlinger S Selection of actions in the basal ganglia-thalamacortical circuits: Review and model International Journal of Psychophysiology 1999 31 3 197 217 10076774 10.1016/s0167-8760(98)00051-8

[B2] DeLong MR Wichmann T Circuits and circuit disorders of the basal ganglia Archives of Neurology 2007 64 1 20 24 17210805 10.1001/archneur.64.1.20

[B3] Nicola S The nucleus accumbens as part of a basal ganglia action selection circuit Psychopharmacology 2007 191 3 521 550 16983543 10.1007/s00213-006-0510-4

[B4] Graybiel AM Rauch SL Toward a Neurobiology of Obsessive-Compulsive Disorder Neuron 2000 28 2 343 347 11144344 10.1016/s0896-6273(00)00113-6

[B5] Terman D Rubin JE Yew AC Wilson CJ Activity patterns in a model for the subthalamopallidal network of the basal ganglia The Journal of Neuroscience 2002 22 7 2963 2976 11923461 10.1523/JNEUROSCI.22-07-02963.2002 PMC6758326

[B6] Chersi F Mirolli M Pezzulo G Baldassarre G A spiking neuron model of the cortico-basal ganglia circuits for goal-directed and habitual action learning Neural Networks 2013 41 212 224 23266482 10.1016/j.neunet.2012.11.009

[B7] McCarthy MM Moore-Kochlacs C Gu X Boyden ES Han X Kopell N Striatal origin of the pathologic beta oscillations in Parkinson's disease Proceedings of the National Academy of Sciences 2011 108 28 11620 11625 10.1073/pnas.1107748108 PMC3136295 21697509

[B8] Marreiros AC Cagnan H Moran RJ Friston KJ Brown P Basal ganglia cortical interactions in Parkinsonian patients NeuroImage 2013 66 301 310 23153964 10.1016/j.neuroimage.2012.10.088 PMC3573233

[B9] Yucelgen C Denizdurduran B Metin S Elibol R Sengor NS A biophysical network model displaying the role of basal ganglia pathways in action selection Artificial Neural Networks and Machine Learning ICANN 2012 7552 177 184

